# Exploring the use of topological data analysis to automatically detect data quality faults

**DOI:** 10.3389/fdata.2022.931398

**Published:** 2022-12-05

**Authors:** M. Eduard Tudoreanu

**Affiliations:** Department of Information Science, University of Arkansas at Little Rock, Little Rock, AR, United States

**Keywords:** topological data analysis, Morse-Smale complex, entity resolution, unsupervised, distance-based point cloud

## Abstract

Data quality problems may occur in various forms in structured and semi-structured data sources. This paper details an unsupervised method of analyzing data quality that is agnostic to the semantics of the data, the format of the encoding, or the internal structure of the dataset. A distance function is used to transform each record of a dataset into an n-dimensional vector of real numbers, which effectively transforms the original data into a high-dimensional point cloud. The shape of the point cloud is then efficiently examined *via* topological data analysis to find high-dimensional anomalies that may signal quality issues. The specific quality faults examined in this paper are the detection of records that, while not exactly the same, refer to the same entity. Our algorithm, based on topological data analysis, provides similar accuracy for both higher and lower quality data and performs better than a baseline approach for data with poor quality.

## Introduction

Modern society is more and more dependent on data and information, and important resources and expertise have been dedicated to managing and improving the quality of data because high quality data can better serve the needs of enterprises, government and people. Data cleaning often requires human expertise and supervision, which is not sustainable in an environment in which the volume, complexity, and velocity of data keeps increasing. The future may require techniques for data cleaning that rely a lot more on automation than on human expertise, with some authors talking about a data washing machine (Talburt et al., 2020) where “dirty” data is cleaned by just turning a few buttons. An organization, such as a corporation or government entity, produces data in many forms, from already curated databases to tables generated internally by departments and individuals, and even through basic email communication. The same organization may also interact with data streaming from the outside, such as news streams, social media, and clients. In order to extract value out of all this information, the organization must first curate the data before integrating it into its business processes. A first step in curation is the ability to automatically identify portions of the data that due to various data quality problems make reference to the same entities, such as persons, parts, or events, but that appear slightly different at a first glance.

This paper explores the use of topological data analysis (TDA) as a means of detecting data quality faults, focusing especially on the existence of duplicates. Topological data analysis (Wasserman, 2018) denotes a number of approaches that rely on examining the shape of various structures present in a multidimensional space to either obtain a simpler view of the shapes or to identify relationships among features of the shapes. Persistent homology, manifold identification, and tree or graph linking (Carlsson, 2014) of the features are all approaches that fall under TDA. TDA has been employed as both a way to detect important patterns in data, but also as a way of reconstructing missing information (van Veen, 2019).

The mathematical characteristics of TDA assumes the space in which the data exists to be a multidimensional coordinate with real numbers, while many computer data sets are often composed of text and numbers. Sometimes even the numbers that appear in a database do not have an algebraic meaning, but are rather identifiers for various entities such as postal codes or phone numbers.

This paper introduces a method of transforming a dataset into a multidimensional point cloud of real numbers that is based on calculating the distance between a subset of selected records {*V*_1_, *V*_2_, …, *V*_*n*_} in the dataset and all other records. The size of the subset determines the number of dimensions for each point in the cloud, and thus the number of dimensions of the space in which topological data analysis takes place. The first record in the subset *V*_1_ is used to compute the first dimension for all the records, the second *V*_2_ to compute the second dimension, and so on (the order of the dimensions 1...*n* does have an impact on the analysis). Three main advantages of our approach are the ability to parallelize the computation of the point cloud, the ability to adjust the dimensions (*n*) to fit allotted time requirements, and the ability to work in an agnostic manner with most types of data. Each dimension of the point cloud can be computed at the same time with other dimensions of the cloud because they do not require much synchronization; the distance between the first record *V*_1_ in the subset and any other record is independent of the distance between the second record *V*_2_ and any other record. The number of dimensions, *n*, can be adjusted on the fly based on how long it takes to compute each dimension. Some precision can be sacrificed if time to completion is more important. Furthermore, additional dimensions can be added at any time due to the intrinsic independence of dimensions.

We want to keep the assumptions about the dataset to a minimum to decrease expert interventions in the data quality detection system. Our solution does not depend on knowing name-value pairs for the fields in a record, and we regard the records almost as sentences in natural language in the sense that each record may have a variable number of “words,” inversions, and inconsistencies (as one would find in PDF files, emails, or online postings). The only assumptions in this paper are that the data is a set of records, and that each record has a variable number of tokens. For the analysis in this report, the tokens are all assumed to be text, and no other assumptions are made about the data type. Although computational TDA methods are part of artificial intelligence, the technique used here does not rely on any supervised learning. Finally, the results in this report do not use any precomputed dictionary of stop words, common misspellings, or abbreviations. Note that such dictionaries could be used as a preprocessing step with our method, and one may assume that they could reduce the noise in the data and lead to better results. However, because this experiment tried to reduce assumptions about the data, the use of dictionaries was ruled out because it would need to consider domain knowledge (for example, “dr” may mean doctor or drive depending on the domain).

Our approach is comparable to more traditional approaches that rely on neural networks for word embedding and clustering algorithms to group records. Both traditional and this approach requires the manipulation of parameters to obtain the best result. The advantages of our approach are (a) that it can be better run in parallel; (b) that TDA supports “explainable AI” because it offers better explanation of the reasons features in the multidimensional space are grouped together: “In fact, many TDA methods may be regarded as visualization methods” (Wasserman, 2018); and (c) it could potentially be used to identify the “shape” of other data faults such as inconsistent representations or missing data and, even further down the road, to automatically correct faults.

## Related work

In the development of a data washing machine, one requirement that occurs in practice often is the need to identify non-exact duplication, otherwise known as entity resolution. Talburt et al. (2020) introduce the idea of a data washing machine, and describe a proof of concept that uses entropy and iterative creation of blocks to achieve this result. Our approach is complementary to theirs in that we explore alternative solutions based on topological features to detect, and in the future to even correct, duplicates.

Solutions for entity resolution rely often on growing pairwise correlations into more complete clusters that represent the same entity. Draisbach et al. (2019) compare and contrast many such approaches, including three new ones they propose. Other authors experiment with various features of clustering techniques, such as adaptive matching (Cohen and Richman, 2002), fast approximate distance measures (McCallum et al., 2000), microclustering (Betancourt et al., 2016), similarity of joined sets (Ribeiro et al., 2018), genetic programming (Yuvaraju and Nivedita, 2013), incremental clustering (Vatsalan et al., 2020), or ordinal regression (Yan et al., 2020). This paper does not focus on clustering pairs of records, but relies on topological features to determine areas of a multidimensional space in which similar records reside. As mentioned before, TDA offers a number of advantages from a researcher's perspective in that it is better suited to provide an explanation of why the computer made its decision and it could be used in future work to focus on automatically identifying other data quality issues beyond duplicate entries and even on automatic data curation.

Pairwise comparison of records is not practical for millions or tens of millions of records, thus solutions involving the breaking down of the search space into blocks (also known as blocking) can significantly reduce the number of operations needed. This set of techniques can be applied before our solution to restrict the size, and thus the topological shape of each detected block. Blocks can be refined iteratively (Papadakis et al., 2014; O'Hare et al., 2019), recursively (Yu, 2020), progressively through a classifier (Galhotra et al., 2021), or probabilistically (Wang et al., 2016; Enamorado and Steorts, 2020). A technique combining a number of blocking pipelines is JedAI (Papadakis et al., 2020) which provides for both schema-based and attribute-based matching, as well as a computational budget to perform the entity resolution. Some blocking techniques are unsupervised, but they do rely on at least a name-value pair relationship (JedAI) if not an outright schema knowledge. The name-value pair is a stronger requirement than our variable list of tokens for each record. Finally, as mentioned above, TDA has the potential for advancing beyond entity resolution.

Machine learning is becoming important in the automation of entity resolution. Techniques span from logistic regression (Kobayashi et al., 2018; Ye and Talburt, 2019), support vector machines (Christen, 2008), decision trees (Warnke-Sommer and Damann, 2019), conditional random fields and word embeddings (Comber and Arribas-Bel, 2019), crowdsourcing the initial learning (Chen et al., 2018), and ensemble solutions (Chen et al., 2009). Deep neural networks are also used with various levels of training (Loster et al., 2021) in addition to the fine-tuning of BERT (Li et al., 2021). Most of the approaches assume the data is structured and require human intervention in an initial phase of the learning process, unlike our solution which focuses on purely unsupervised detection. One solution based on gradual machine learning (Hou et al., 2019) replaces training by a human expert with an iterative build-up of annotations.

An unsupervised approach for entity resolution applied to authors of articles is presented in Dai and Storkey (2011) and focuses on a hierarchical model that generates agglomerative clusters without the use of parameters. Another bibliographic-based data set that is unsupervised and generative is described in Bhattacharya and Getoor (2006). The type of data in these particular cases is focused on authors and coauthors of papers unlike the more general data sets that we want to focus on and which are needed for a data washing machine. Another unsupervised approach (Kirielle et al., 2022) relies on graphs and can deal with more complex entities, but their solution runs under the assumption that schema is known, for example that certain records are birth information and others are marriage.

## Duplicates detection algorithm

At its core, the algorithm uses Morse Theory (Forman, 2002) to efficiently find partitions of a high dimensional space defined *via* a point cloud. The points in each partition have similar features, and therefore can be considered similar. Each point in the cloud corresponds to a record in the dataset, and points within the same space partition have a good chance of being duplicates.

### Step 1: Preprocessing

The input to our algorithm is assumed to have little structure, similar to either a comma-separated or tab-separated text file. Records are assumed to be separated by new lines, and each record can have a variable number of fields, or tokens. The algorithm assumes missing fields, or swapping of fields from one row to the next, and as such, if a header row exists, it will be ignored. Tokens are separated by comma or white space characters. All other separator characters such as period, dash, or slash are assumed to be part of the same token, and they are deleted, resulting in an aggregator of the content of the token. For example, the token “555-1212” becomes “5551212”. Another possibility in this preprocessing step would be to separate the two sets of digits into two tokens “555” “1212”.

Parameters available to a user at this step:

1. Determine whether a header row exists or not. Possible values are yes or no.
2. Choose aggregation or splitting of tokens when a separating character is encountered. Possible values are aggregate or split.

### Step 2: Point cloud generation

The solution we chose relies on selecting a subset of records to be used almost as viewpoints in the dataset space. The subset can be chosen iteratively, that is not all at once, and can be extended if needed.

Our solution includes a method of deriving a point cloud to encode a set of arbitrary records in a dataset to an n-dimensional Real-number domain. The process relies on selecting a set of points {*V*_1_, …, *V*_*n*_} which are used to compute each dimension n. Technically, a distance is computed between *V*_*i*_ and every other record, for all *i* between 1 and *n*. In this manner a *n*-dimensional point is determined for each record, and that point is the n distances between the record and *V*_1_ through *V*_*n*_. Intuitively, each point *V*_*i*_ serves as a viewpoint from which the rest of the records are observed; an analogy in 2D space may be picking various people inside a meeting room and recording each person's perspective. The more spread out the viewpoints are, the more hidden “details” can be viewed.

Approaches for selecting viewpoints, *V*_*i*_, span from completely random, to completely deterministic and in-between. Selecting *V*_*i*_ at random has the advantage of allowing each of the *n*-dimensions to be computed in a highly parallel fashion with very little synchronization between threads. At the other end of the spectrum, after initially selecting *V*_1_ at random, the next record *V*_2_ can be the one that is at the furthest distance from *V*_1_. Similarly, *V*_3_ would be selected to be the furthest from *V*_2_, not including *V*_1_. This can be repeated for all records all the way to *V*_*n*_. This is the method implemented for this paper. An in-between solution would be to have *n* = *k* ^*^ *s*, and use a random selection for the first *k* points, followed by a deterministic, distance-maximizing selection of s points using the first *k* randomly-picked records.

The final requirement for our approach is to be able to determine the distance between two arbitrary records, A and B. We adapted the Damerau–Levenshtein (Damerau, 1964) distance to consider records of tokens. A record is viewed as a sequence of tokens, and each token is a string of characters (or symbols). For example, records A and B are:

- A: JOHN DOE MAIN ST
- B: JON DOE ST MIAN

where the tokens are JOHN, JON, DOE, MAIN, MIAN, and ST. Because each token is a string, the Damerau–Levenshtein distance is directly applicable when looking at tokens alone. As such, the distance between MAIN and MIAN would be 1, representing one swap operation of I with A.

An entire record is viewed as a sequence of tokens, just as a string is a sequence of characters. We treat tokens as if they were individual characters, and run something similar to Damerau–Levenshtein to see if a token needs to be deleted, inserted, replaced, or its position swapped with its neighbor. The main difference is that the cost of deletion, insertion, and swap can be an integer larger than 1 (in the case of strings, when dealing with a single character, the cost of an operation is either 0 or 1). For example, the cost to insert or delete the token JOHN is four because of its four characters. The cost to replace DOE with JON is two because D would need to be replaced by J and E by N.

Once tokens are viewed as characters, all possibilities of inserting, deleting, replacing, and swapping positions are considered, which makes the calculation of the distance between two records proportional to the square of the maximum number of tokens in any record.

One condition that is different when considering tokens rather than single characters is that swapping of two tokens should only be considered when the two tokens are relatively close. The original Damerau–Levenshtein distance would allow swapping of two characters, for example IA in one string and AI in the other, only if the characters are exactly the same. We relax that exact-match requirement, and allow for two tokens to be considered for swapping if their Damerau–Levenshtein distance is below a threshold. That threshold is captured by a parameter in our algorithm, *maximum swap distance*, which limits swapping calculations to tokens whose distance from each other is no larger than *maximum swap distance*. A maximum distance of zero would require an exact match for the tokens. The pseudo-code to compute the distance between two records rec1 and rec2, where each record is an array or tokens (or strings), uses recursion and is included below for reference:


//compute the distance between two records with the following call
compute_distance(rec1, rec2, len(rec1) - 1, len(rec2)
     - 1, max_swap_distance)
//definition of the function
function compute_distance(rec1, rec2, *i*, *j*,
     max_swap_distance)
   if *i* == -1 and *j* == -1 //no token left to process
        return 0
   if (*i*, *j*) in tmpMem //tokens rec1[*i*] and rec2[*j*] have already been done
        return tmpMem[(*i*, *j*)]
   if *i* $>$ $-$1 //there are still tokens in rec1
      tmp1 = compute_distance(rec1, rec2, *i* - 1, *j*, max_swap_distance) +
         len(rec1[*i*])
   if *j* > -1 //there are still tokens in rec2
      tmp2 $=$ compute_distance(rec1, rec2, *i*, *j* -1, max_swap_distance) +
         len(rec2[*j*])
 tmp = min(tmp, tmp2)
 if *i* > -1 and *j* > -1 //there are tokens left in both records
      tmp3 = compute_distance(rec1, rec2, *i* - 1, *j* - 1, max_swap_distance) +
            damerauLevenshtein(rec1[*i*], rec2[*j*])
  m = max(damerauLevenshtein(rec1[*i*], rec2[*j* -~1]),
            damerauLevenshtein(rec1[*i* -- 1], rec2[*j*]))
 if *i* > 0 and *j* >0 and \textit{m} <
   max_swap_distance
 //see if they are close enough, by distance \textit{m}, to consider a swap
      tmp4 = compute_distance(rec1, rec2, *i* - 2, *j* - 2) + \textit{m}
 tmpMem[*i*, *j*] = min(tmp1, tmp2, tmp3, tmp4)
 return tmpMem[*i*, *j*]


Parameters available to a user at this step:

3. Dimensions to be considered for each record. Possible values are from 1 to *M* – 1, where *M* is the total number of records in the data.
4. Maximum swap distance. Possible values are integers from 0 (meaning no swapping allowed), to max_length, where max_length is the length of the longest token in the data set (meaning the order of tokens in the records is completely ignored).

#### Word embedding vs. our distance function

Another solution available to us would have been to use a neural network to generate an embedding for each record (Mikolov et al., 2013), similar to *doc2vec* (Gensim, 2002). We decided against that approach for three reasons: (a) the difference between records can more easily be parallelized, (b) the use of both neural networks and TDA, both of which are part of artificial intelligence, would have made it difficult to judge the performance of TDA alone, and (c) pursue more easily explainable AI by avoiding embedding solutions, which rely on neural networks whose decisions are not easily explainable to a human analyst.

**Figure 1:**
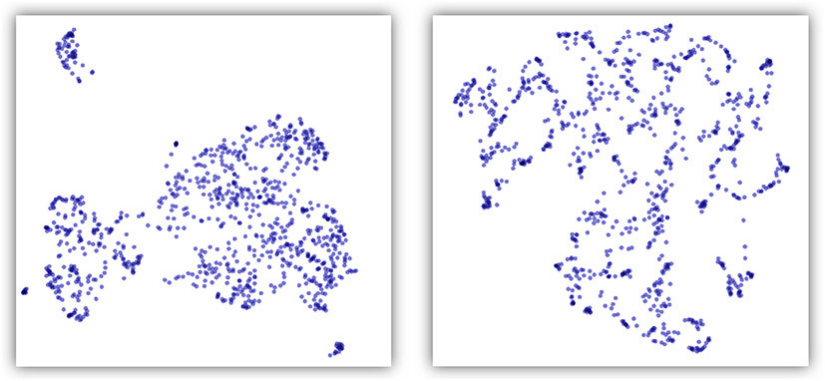
Comparison of point clouds obtained with our distance method **(left)** and *doc2vec* embedding **(right)**. The dimensionality of the point cloud space was reduced to 2 using UMAP. The data set presented here is the poor quality one with 1,000 records.

The shape of the point cloud created by word embedding also appears different from the one created by our approach. Both are high-dimensional spaces, and we examined them visually using a number of dimension reduction techniques. As shown in Figure 1, with UMAP (McInnes et al., 2018), also a TDA-based solution, word embedding appears as intersecting strands while our distance-based point cloud resembles more evenly spaced blobs and clusters.

### Step 3: Morse-Smale complex calculations

Topological data analysis refers to a number of mathematical approaches (Wasserman, 2018), but for this article we relied on Morse-Smale complexes to find records, in the form of point clouds, that exist in the same areas of a multidimensional space. Morse Theory studies the structures of a manifold, an n-dimensional topological space, by considering decomposition and homology. Homology allows complex manifolds, for example one that may have observation noise, to be viewed as simple equivalent ones, which in effect can ignore some of that noise and provide relevant information about their structure.

**Figure 2:**
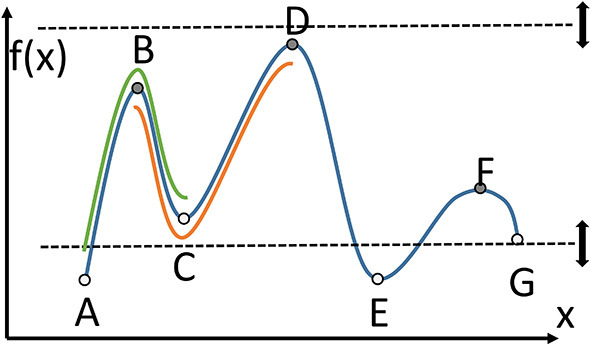
Important concepts of Morse theory for a 1D function. Critical points, local minima and maxima, A through F. Ascending manifold BCD anchored at C. Descending manifold ABC anchored at B. Morse-Smale complex BC, intersection of the two manifolds.

For a simpler presentation, consider Figure 2 that shows a 1D function, and thus a 1D manifold, going through all the points A through F. There are some critical points to consider, and those include minima, maxima, and equivalents in higher dimensional space, for example saddle shapes in 2D. There are two types of manifolds, an ascending manifold, shown from B to C to D under the main function. That manifold is anchored at a minimum, C. Similarly there are descending manifolds, and one from A to B to C is highlighted anchored at a maximum, B. A Morse-Smale complex is the intersection of an ascending and a descending manifold (under certain conditions), and it can be seen in Figure 2 from B to C.

**Figure 3:**
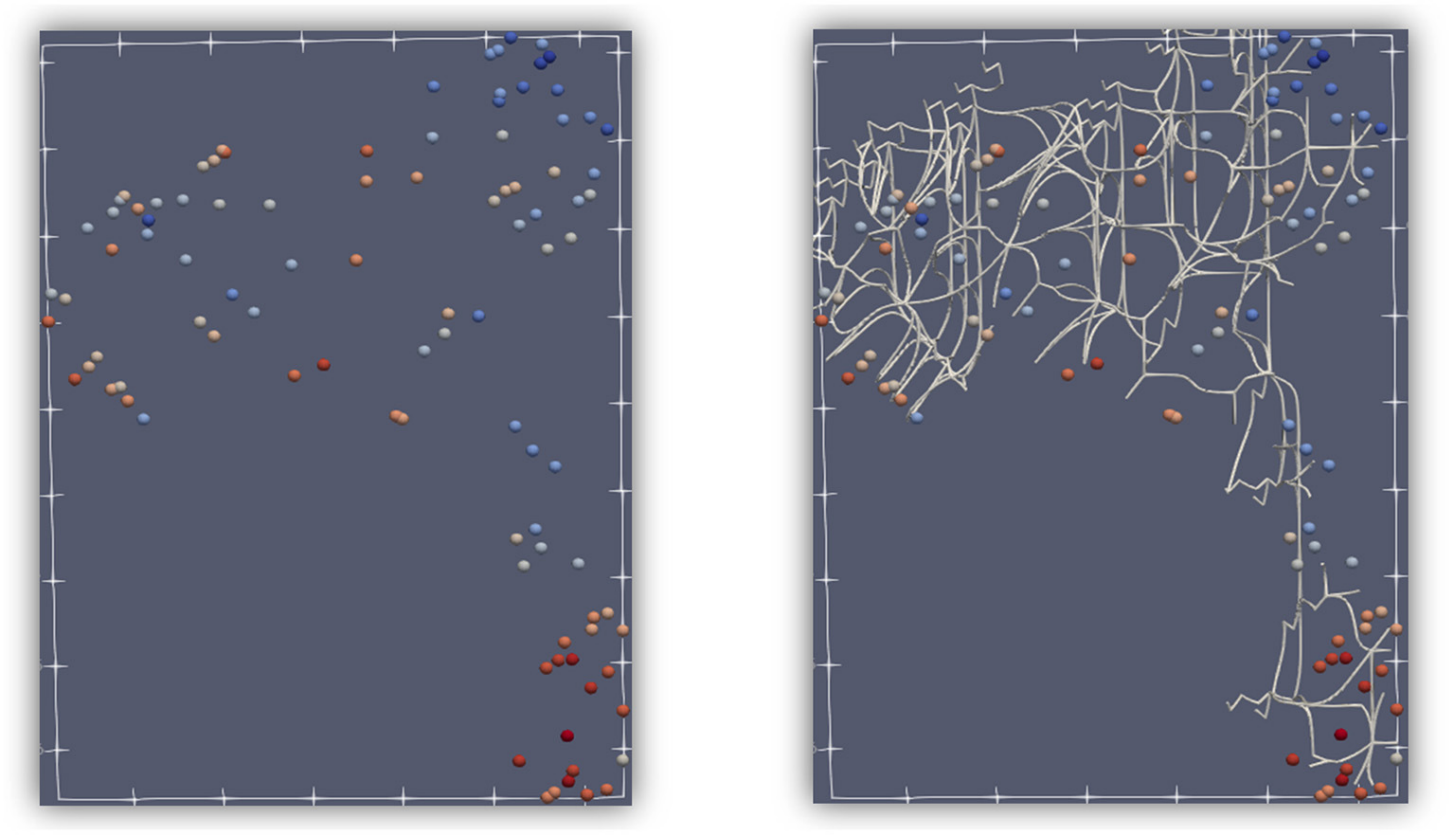
An example of how 2-separatrices can provide a division of a 2-dimensional space. A dataset of points shown in 2 dimensions **(left)**, with similar color symbolizing similar features. The same dataset is divided by 2-separatrices **(right)**. Notice that points with about the same color tend to be captured inside the areas defined by separatrices, which is an illustration of Morse theory.

Another aspect of TDA includes persistent homology, specifically the notion of persistence. Critical points can change when only a subset of the function is considered. In Figure 2, the dashed lines are used to restrict the function to those points that exist only between the upper and lower dashed lines. As those lines are moved up and down, some critical points are no longer considered, or some local minima or maxima are no longer the absolute minimum or maximum. In the figure, C and G are critical points, and A and E are not yet critical. A and E will become critical when the lower dashed line moves even lower. As the dashed lines move, some critical points stay critical longer than others, which is at the basis of persistence. Another important feature that is used in TDA is the connection between points that become critical or cease to be critical at about the same “time” (“time” is controlled by movement of the dashed lines). These connections form a graph or binary relationships between points, and that can be used for geometry simplification. Critical points can be connected by separatrices, which divide n-dimensional space. Figure 3 shows the division of 2D space formed by a point cloud of 100 records, which is one of the datasets used later in the paper. By considering critical points at different persistence levels, different sets of separatrices, such as the white lines in Figure 3, can be brought into the calculations and into the division of the space.

On the technical level, our algorithm uses the *topopy* (Maljovec, 2020) package to compute the Morse-Smale complexes. The function that computes the Morse-Smale complex takes two arguments, the point cloud of records and a scalar value; the value given is the constant 1 for each point. Two parameters are important for our purposes: the point graph *beta* which can be viewed as providing a measure of the attraction of points in the graph used by *topopy*, and a *normalization* algorithm type that has two values “zscore” and “feature.” “Zscore” ensures that the data has a mean of zero and a standard deviation of 1. “Feature” scales the data inside the unit hypercube.

Parameters available to a user at this step:

5. Beta: controls how many Morse-Smale complexes are created and how large those complexes are; fewer complexes leads to more points/records on average assigned to each complex. Possible values are positive, non-zero numbers, but the relevant range would be determined by the distance between the points in the high dimensional range. Outside of that range, either each Morse-Smale complex has a single point or all the points are placed inside a single complex.
6. Normalization: either “zscore” or “feature”.

## Algorithm evaluation

In this section, we compare our algorithm with the baseline of using word embedding and a popular clustering algorithm, *dbscan* (dbscan, 2022). This baseline was also used by other authors such as Talburt et al. (2020). The more modern Hdbscan (Campello et al., 2013) was also tried as a baseline, but it performed slightly worse than the original. Note that Magellan (Konda et al., 2016), a popular baseline used for example by Li et al. (2021) and Kirielle et al. (2022), and partially by JedAI (Papadakis et al., 2020), does not support “dirty” data and, thus cannot be used for this evaluation.

The datasets employed in this experiment were synthetically generated using the SOG method established by Talburt et al. (2009) which allows control over the quality of the records and provides uniformity throughout the dataset. SOG uses its seed data, such as name and address, from publicly available domicile occupancy records from several states in the U.S., resulting in name and address distribution close to the actual distribution in those states. The address structure is similar to real addresses and is intended to pass commercial verification tools. Synthetic persons are generated, attached to addresses, and given additional features such as birth dates, phone numbers, and so on. SOG can be programmed to generate various levels of data faults, and to simulate the same synthetic person moving to a different location. This method was chosen because it provides data that has the same structure and distribution as real-world data, but with the additional benefit of the absolute truth already known with 100% accuracy. Furthermore, as noted by Kirielle et al. (2022) “in the context of databases that contain complex entities such as person records, ground truth data are often not available, or if available they might be limited to manually curated, biased, and incomplete matches.”

We generated a “good” dataset of size 100 that has relatively few quality problems, henceforth referred to as G100, and a poor quality data set of size 1,000 (P1000). Samples from the two datasets are given below. The target structure of an ideal record is listed at the top of each sample as a reference for the reader. Our experiment ignores the information in that first row, and both G100 and P1000 have quality faults such as misspelling, missing field, inverted fields, and inconsistent format.

G100:

- RecID,fname,lname,mname,address,city,state,zip,ssn
- A944634,IAN,AADLAND,LARS,29021 HIGH SIERRA TRL,SANTA CLARITA,CA,91390,490-46-2048
- A755471,MYRA,AARGAARD-ESPERSEN„1224 MAGNOLIA ST,WINSTON SALEM,NC,27103,117-15-8521
- A912696,MYRA,AAARGAARD-ESPERSEN„1224 MAGNOLIA ST,WINSTON SALEM,NC,27103,117158521
- A813025,ALLEN,AARON,IKAIKA,3830 COUNTRY CLUB RD # J,WINSTON SALEM,NC,27104,

P1P000:

- RecID,Name,Address,City,State,Zip1,City,State,Zip2,PO, Box,POCity1,State,Zip,POCity2,State,Zip,SSN,DOB
- A912969,barbaar,myers-christian,3536,N,BERLIN,AVE,FRESNO„CA,93722,Po, Box,5991,fresno„ca,93755,010-52-5974,1936
- A915784,b,chavez,9247,TOBIAES,AVE,PNORAMA,CITY„ CALIFORNIA,91402,pomst,office,4601,Panorama,City„ California,91412,10525974,1936
- A933400,barby,chavez,11881,GULF,POINTE,DR,APT,E38, HOUSTON„TX,77089„,010-55-2974,19360814,
- A968925,CHAVEZ,2943,N,COTTONWOOD,ST,WUNIT, 3,Orange„Ca,92865„„,

There are a number of parameters to consider for both the baseline and our solution. While some of those were chosen and kept constant during our tests, other parameters were varied through a small number of combinations to achieve the best result in terms of accuracy and F-value. The goal was to determine what can be achieved quickly by just turning a few “knobs” of the data washing machine, rather than to go through a detailed and deliberate variation of many possible parameter values in order to maximize some set criteria.

The baseline test implemented a two step process of first computing a multidimensional number for each record and, then, clustering those numbers based on how close they are to each other in that multidimensional space. We used *doc2vec* and *dbscan*, both available from python repositories. The first program uses a neural network to create an embedding, that is a sequence of numbers, for each record. The second performs clustering on that sequence of numbers by regarding each record as a point in a multidimensional space. The parameters used for this test, which are in line with other experiments (Talburt et al., 2020), are:

- *doc2vec*: dimensions = 5, min_count = 2, epochs = 500, alpha = 0.25, min_alpha = 0.00025; note that we experimented with higher dimensionality, but the results were significantly worse than the chosen number of 5;
- *dbscan*: min_samples = 1, eps = 1.5, which gave the best result for the G100 file, and 2.25, best result for the P1000 file.

**Figure 4:**
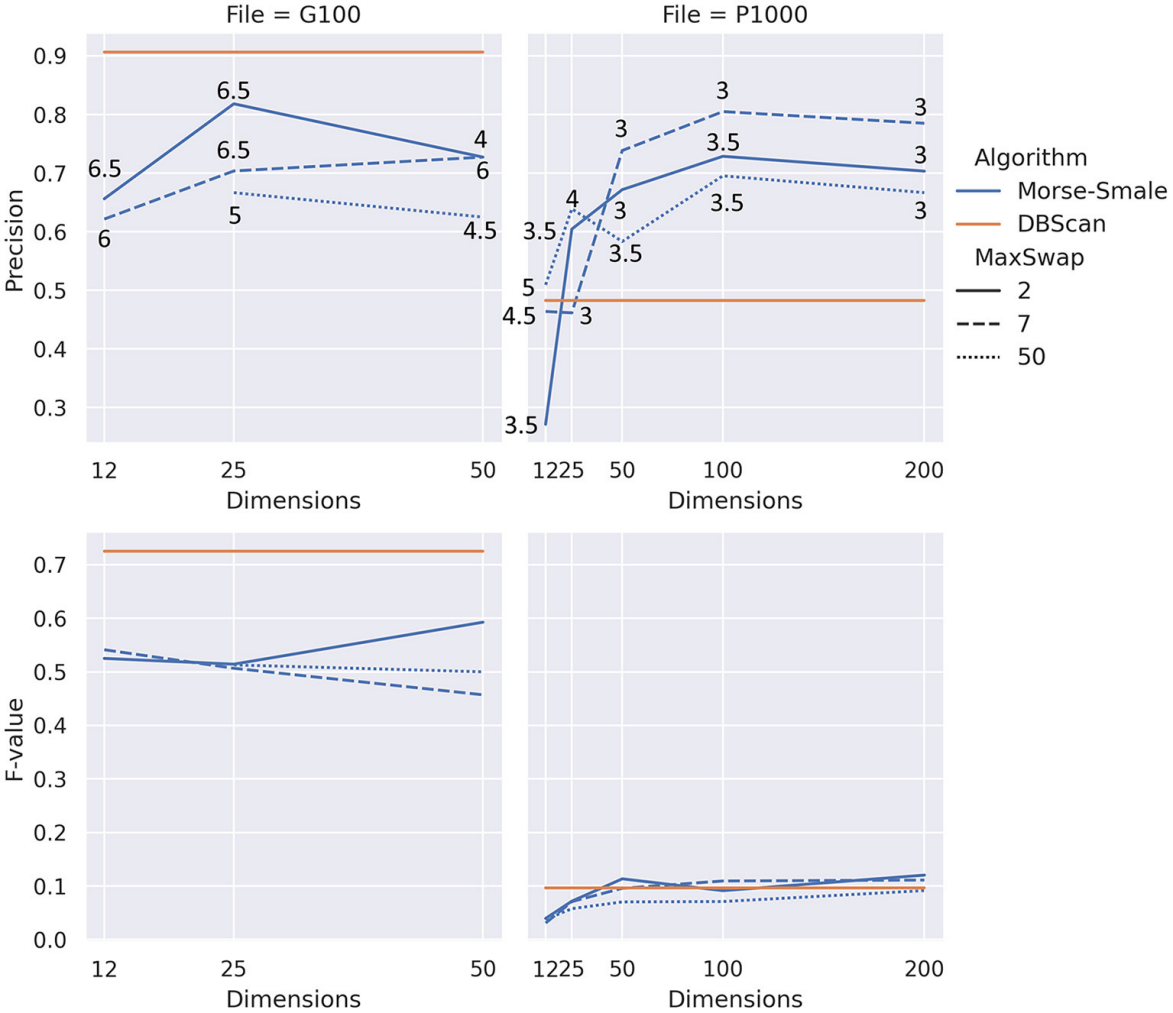
Results of our experiment showing precision **(top)** and *F*-value **(bottom)** for two different files, good quality **(left)** and poor quality **(right)**. The numbers on the top plots show the values of beta that yielded the best result. Note that *dbscan*, horizontal straight lines, are shown as a reference for the precision and *F*-value, with no relation to the number of dimensions (x-axis).

For our algorithm, the focus of this paper, we ignored the header row, used aggregation inside a token, and used the “zscore” normalization for Morse-Smale complexes. The number of dimensions in the experiment followed an exponential scale with the values 12, 25, 50, 100, and 200. The last two values were only considered for the larger file P1000, which has 1,000 records. As for the maximum swap distance, we considered three values for the distance: 2, which is restrictive in that it allows swapping of tokens that are almost identical, 7 which permits tokens that are more different from each other to be considered for swapping, and 50 which will allow most pairs of tokens to be considered for swapping. The sixth, and final, parameter to consider is beta, which was varied to achieve the best accuracy and F-value. We only considered a small subset of beta values from 3 to 7, with an increment of 0.5. Different beta values gave the best result for different files, dimensions, and maximum swap distances. Figure 4 shows the results visually for both the precisions (i.e., accuracy) and *F*-value.

## Results

Our algorithm performs better than the baseline for data with lower quality (P1000 in Figure 4), but it does worse than the baseline for data with better quality. Precision for our approach seems to be in the same range regardless of the quality of the data. On the other hand, *F*-value decrease significantly when the data quality decreases for both the baseline and our algorithm.

In most conditions from Figure 4, increasing the number of dimensions of the point cloud space appears to reach a saturation value after which the precision starts to drop. Too few dimensions, 12 in this experiment, can also lead to low precision.

The maximum swap distance has an effect on the precision of our algorithm. Allowing most tokens to be swapped, a distance of 50, is consistently less accurate than smaller maximum swap distances. Values in the interval 0 through 7 may be more appropriate based on the conditions tested in our experiments. Different data quality problems may be better suited to different values. If inconsistent abbreviations and re-ordering of tokens is observed, then values at the higher end of the interval are likely to work best.

As a general behavior, lower beta values tend to result in more (not necessarily correct) duplicates being detected by our algorithm. After reaching a peak *F*-value, further increase in beta value appears to result in better precision and worse *F*-values because the algorithm detects fewer pairs of records, but those detected are more likely to be actual duplicates. Note that *dbscan*, the baseline, exhibits a similar behavior with respect to its eps parameter.

## Conclusions and future work

We presented an algorithm for finding duplicates in an arbitrary dataset that shows promise for data with poor quality. The intention was to avoid performing significant pre-processing and prior cleaning of data. The algorithm relies on TDA and a distance-based point cloud calculation, without the use of any other artificial intelligence solution, such as neural networks. In the future, the TDA may provide the means for unsupervised identification of data quality issues other than duplicate entities and even for automatic reconstruction of a dataset by analyzing the context of duplicates and correcting their information based on the entirety of other records, similar to van Veen (2019).

## Data availability statement

The raw data supporting the conclusions of this article will be made available by the authors, without undue reservation.

## Author contributions

The author confirms being the sole contributor of this work and has approved it for publication.

## Funding

This material is based upon work supported by the National Science Foundation under Award No. OIA-1946391.

## Conflict of interest

The author declares that the research was conducted in the absence of any commercial or financial relationships that could be construed as a potential conflict of interest.

## Publisher's note

All claims expressed in this article are solely those of the authors and do not necessarily represent those of their affiliated organizations, or those of the publisher, the editors and the reviewers. Any product that may be evaluated in this article, or claim that may be made by its manufacturer, is not guaranteed or endorsed by the publisher.

## Author disclaimer

Any opinions, findings, and conclusions or recommendations expressed in this material are those of the author(s) and do not necessarily reflect the views of the National Science Foundation.
